# The Influence of Body Fat Dynamics on Pulmonary Immune Responses in Murine Tuberculosis: Unraveling Sex-Specific Insights

**DOI:** 10.3390/ijms25136823

**Published:** 2024-06-21

**Authors:** Dhanya Dhanyalayam, Hariprasad Thangavel, Tabinda Sidrat, Neelam Oswal, Kezia Lizardo, Michael Mauro, Xin Zhao, Hai-Hui Xue, Jigar V. Desai, Jyothi F. Nagajyothi

**Affiliations:** Center for Discovery and Innovation, Hackensack Meridian Health, Nutley, NJ 07110, USA; dhanya.dhanyalayam@hmh-cdi.org (D.D.); hariprasad.thangavel@hmh-cdi.org (H.T.); tabinda.sidrat@hmh-cdi.org (T.S.); neelam.oswal@hmh-cdi.org (N.O.); kezia.lizardo@hmh-cdi.org (K.L.); mmauro2@ycp.edu (M.M.); xin.zhao@hmh-cdi.org (X.Z.); haihui.xue@hmh-cdi.org (H.-H.X.); jigarkumar.desai@hmh-cdi.org (J.V.D.)

**Keywords:** *Mycobacterium tuberculosis*, acute infection, diet, body fat, fat gain, fat loss, FAT-ATTAC mouse, sex difference, pulmonary pathology, immunometabolic signaling

## Abstract

The World Health Organization (WHO) highlights a greater susceptibility of males to tuberculosis (TB), a vulnerability attributed to sex-specific variations in body fat and dietary factors. Our study delves into the unexplored terrain of how alterations in body fat influence *Mycobacterium tuberculosis* (*Mtb*) burden, lung pathology, immune responses, and gene expression, with a focus on sex-specific dynamics. Utilizing a low-dose *Mtb*-HN878 clinical strain infection model, we employ transgenic FAT-ATTAC mice with modulable body fat to explore the impact of fat loss (via fat ablation) and fat gain (via a medium-fat diet, MFD). Firstly, our investigation unveils that *Mtb* infection triggers severe pulmonary pathology in males, marked by shifts in metabolic signaling involving heightened lipid hydrolysis and proinflammatory signaling driven by IL-6 and localized pro-inflammatory CD8^+^ cells. This stands in stark contrast to females on a control regular diet (RD). Secondly, our findings indicate that both fat loss and fat gain in males lead to significantly elevated (1.6-fold (*p* ≤ 0.01) and 1.7-fold (*p* ≤ 0.001), respectively) *Mtb* burden in the lungs compared to females during *Mtb* infection (where fat loss and gain did not alter *Mtb* load in the lungs). This upsurge is associated with impaired lung lipid metabolism and intensified mitochondrial oxidative phosphorylation-regulated activity in lung CD8^+^ cells during *Mtb* infection. Additionally, our research brings to light that females exhibit a more robust systemic IFNγ (*p* ≤ 0.001) response than males during *Mtb* infection. This heightened response may either prevent active disease or contribute to latency in females during *Mtb* infection. In summary, our comprehensive analysis of the interplay between body fat changes and sex bias in *Mtb* infection reveals that alterations in body fat critically impact pulmonary pathology in males. Specifically, these changes significantly reduce the levels of pulmonary CD8^+^ T-cells and increase the *Mtb* burden in the lungs compared to females. The reduction in CD8^+^ cells in males is linked to an increase in mitochondrial oxidative phosphorylation and a decrease in TNFα, which are essential for CD8^+^ cell activation.

## 1. Introduction

*Mycobacterium tuberculosis* (*Mtb*), a bacterium primarily affecting the lungs, is commonly known as pulmonary tuberculosis (TB) [1,2]. In 2021, an estimated 10.6 million individuals worldwide were reported to have contracted *Mtb*, according to the World Health Organization (WHO) [3]. TB presents a significant global health challenge, and ongoing collaborative efforts involving governments, international organizations, and healthcare providers aim to combat and ultimately eradicate this disease. The global TB pandemic exhibits marked disparities in prevalence between men and women, with a male-to-female ratio of 1.8 in worldwide case notifications [4,5,6,7,8]. Nonetheless, the precise reasons behind these sex-based variations in TB prevalence remain not fully elucidated.

Body weight significantly impacts both the onset and management of TB disease, as well as the treatment outcomes in TB patients. TB can lead to weight loss due to increased energy expenditure and decreased appetite. Previously, we demonstrated that a decrease in body weight resulting from a reduction in body fat increases pulmonary pathology and the risk of TB activation in a low-grade *Mtb* (laboratory-adapted strain *Mtb* H37Rv) infection using a transgenic, inducible “fatless” murine TB model system, the FAT-ATTAC (fat apoptosis through targeted activation of caspase 8) mouse [9]. In particular, we found that *Mtb* persists in fat tissue, and a decrease in body fat leads to increased M2-polarized macrophages in the lungs, resulting in heightened pulmonary pathology during acute infection [9]. Furthermore, our study revealed that an excessive fat accumulation (obese condition) induced by a high-fat diet also exacerbates *Mtb* load and lung pathology in comparison to mice consuming a low-fat diet during acute *Mtb* (H37Rv strain) infection [10]. These observations, in conjunction with other studies highlighting the impact of body fat fluctuations on the pathogenesis of *Mtb* infection, underscore the significant role of body fat in modulating both systemic and local metabolic and immune responses during infections, particularly in the context of *Mtb* infections [11,12,13].

According to the WHO, men worldwide are at a significantly higher risk of contracting and succumbing to TB than women. Prior research in murine H37Rv-infection models has revealed that male and female mice elicit distinct immune responses in the lungs; this discrepancy is associated with a more pronounced pro-inflammatory response and B-cell follicle formation, contributing to increased male susceptibility to *Mtb* infection [14,15]. It is important to highlight the significant role that fat cells play in maintaining overall immune homeostasis, with observed sex-related disparities in body fat levels and distribution patterns [16]. However, whether body fat can distinctly modulate immune cell activation within the lungs of males and females during *Mtb* infection has yet to be explored. Building upon our earlier research, we hypothesize that body fat is a critical determinant in shaping both the systemic and local anti-bacterial immune responses, thereby impacting the lung bacterial load during acute *Mtb* infection. Consequently, these mechanisms differentially influence pulmonary pathogenesis in males and females.

The main objective of our descriptive study is to explore the effects of changes in body fat during acute infection on post-acute lung pathology, including *Mtb* burden, immune responses, and T-cell activation, with a particular emphasis on sex-specific dynamics. In this study, we employed a low-dose of the highly virulent *Mtb*-HN878 clinical strain to infect male and female FAT-ATTAC mice, evaluating them at the 30-day post-infection mark. This time frame corresponds to a sub-acute phase of infection, a critical period that significantly influences the fate of the bacterium and the remodeling of lung tissue. Within this experimental framework, we subjected the mice to two different conditions: some were fed a medium-fat diet (MFD) to promote body fat gain, while others underwent fat ablation (FAB+) to induce body fat loss. To gain insights into the impact of these body fat alterations, whether through increase or decrease, on pulmonary pathology, immune cell infiltration, and the control of bacterial burden, we conducted thorough histopathological and immunophenotyping analyses on lung tissues from both male and female mice. First, our research focused on exploring the variations in metabolic and inflammatory mechanisms in the lungs between males and females fed a regular diet (RD) during acute *Mtb* infections. This includes examining the role of lipid hydrolysis, mitochondrial oxidative phosphorylation, and the IL-6 response. Next, we examined the impact of fat loss and fat gain on pulmonary pathology and *Mtb* burden, and whether these effects vary between males and females. Furthermore, we examined the consequences of fat loss and fat gain on pulmonary CD8^+^ T-cells, assessing their mitochondrial oxidative function and activation. Thus, by analyzing the lung tissues and immune cells, we sought to elucidate the intricate relationship between body fat, immune responses, and *Mtb* infection outcomes, taking into consideration sex-specific differences.

## 2. Results

Previously, we demonstrated that an acute loss of body fat increases the risk of *Mtb* activation by inducing M2 macrophages in the lungs of H37Rv-infected transgenic fat-ablatable FAT-ATTAC mice [9]. Additionally, we showed that a high-fat diet that induces accumulation of excessive fat (consisting of 60% fat) leads to an elevated *Mtb* burden in the lungs of C57BL/6J mice infected with H37Rv [10]. Mice fed with HFD develop obese conditions with insulin resistance [10]. These observations highlight that both the fat loss and excessive fat gain regulate pulmonary pathology and *Mtb* load, suggesting that body fat levels likely play a pivotal role in immune regulation and containment of bacteria during *Mtb* infection. Given that males exhibit greater susceptibility to *Mtb* infection, and their body fat levels, and distribution patterns differ from those of females, we hypothesize that a moderate increase in body fat and an acute fat loss will have distinct effects on pulmonary pathology, including the activation of immune cells, in both males and females during acute *Mtb* infection. During this study, conducted in the sub-acute phase of *Mtb* infection, our primary objectives were to examine the consequences of changes in body fat on the following aspects: (i) Impact lipid metabolism in the lungs, influencing *Mtb* gene expression and affecting the pulmonary bacterial burden. (ii) Influence the presence and activation of immune cells in the lungs. (iii) Affect the metabolism and activation of resting CD8^+^ T-cells in the lungs. (iv) Differentially regulate lung pathology, including bacterial burden, immune cell infiltration and activation, as well as the levels of effector CD8^+^ T-cells in both male and female subjects. To address these objectives, we conducted experiments using age- and sex-matched FAT-ATTAC mice infected with 100 colony-forming units (CFUs) of the *Mtb* HN878 strain, a highly virulent clinical strain. The mice in our study were divided into different groups: a medium-fat diet (MFD) to induce body fat gain, fat ablation (FAB+) to induce body fat loss, and a control group fed a regular diet (RD), as shown in Supplementary Figure S1. Our data analysis was conducted in two steps: (a) comparing the infected RD-fed group with the uninfected RD-fed group and examining sex-specific differences during acute *Mtb* infection; (b) comparing the infected RD-fed group with the infected fat-loss (FAB+ RD-fed) group and fat-gain (MFD-fed) groups to evaluate the effects of fat loss and fat gain on lung pathology in both males and females during the infection.

### 2.1. Effect of Fat Loss and Fat Gain on Body Weight

Clinical reports suggest a correlation between underweight status and increased mortality in individuals with pulmonary TB, while overweight status appears to confer beneficial effects [17]. Previously, we documented a significant reduction in visceral fat mass due to *Mtb* infection, with induced fat ablation further exacerbating visceral body fat loss in H37Rv-infected FAT-ATTAC mice [9]. However, the previous study did not explore the influence of sex on the impact of body fat loss or gain on body weight during *Mtb* infection. Here, we conducted separate analyses to investigate the impact of body fat loss or gain on body weight in male and female mice, both uninfected and infected with the HN878 strain of *Mtb*, both before and after 30 days post-infection (DPI). In general, age-matched male mice exhibit greater body weight compared to female mice [18]. Across genders, male mice consistently exhibited significantly greater body weights (5–15% greater, except one or two mice) both at 0 DPI and 30 DPI compared to females, irrespective of infection status or diet conditions (Supplementary Figure S2). Notably, infected mice generally showed decreased body weights compared to uninfected counterparts, regardless of diet composition, with this effect observed predominantly in males across both regular diet (RD, ≈6% decrease, 22.3 ± 2.6% g vs. 23.6 ± 2.0% g; *p* = 0.0099) and medium-fat diet (MFD, ≈10% decrease, 24.5 ± 1.8% g vs. 27.4 ± 1.7% g; *p* = 0.0000229) conditions, while in females, it was significant only in MFD-fed (≈6% decrease, 21.6 ± 2.2% g vs. 22.9 ± 2.8%; *p* = 0.0135) mice (Supplementary Figure S2A,B). When comparing different infected groups, in males, the loss of body fat through fat-ablation significantly reduced body weight (19.9 ± 3.3% g, ≈11% decrease; *p* = 0.00067), whereas the gain of body fat through MFD feeding significantly increased (24.5 ± 1.8% g, ≈10% increase; *p* = 0.00037) body weight compared to infected mice fed an RD (22.3 ± 2.6% g) (Supplementary Figure S2C). Conversely, in females, fat-ablation did not yield a significant alteration in body weight among infected mice, while MFD feeding led to a significant increase in body weight (21.6 ± 2.2% g, ≈10.5% increase, *p* = 0.014) compared to RD-fed counterparts (19.5 ± 6.3% g) (Supplementary Figure S2D). These findings suggest that *Mtb* infection and induced fat-ablation exert distinct regulatory effects on body weight in male and female subjects within a murine TB model.

### 2.2. Fat Loss and Fat Gain Alter Lung Pathology in Mtb Infection

**Figure 1:**
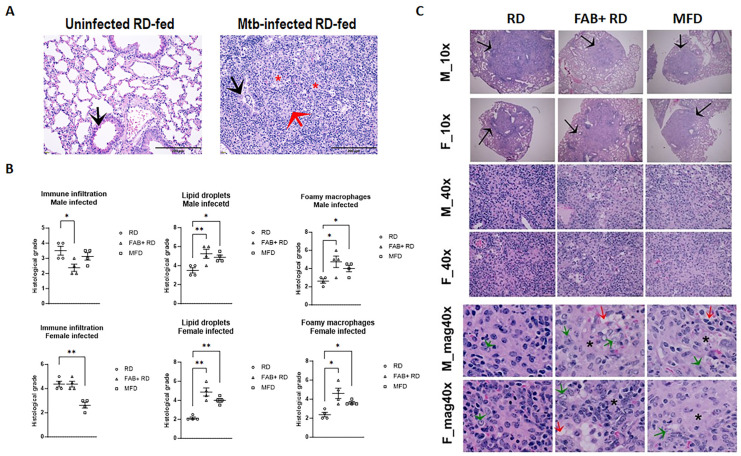
Fat gain (MFD) and fat loss (FAB+) alter lung histopathology during HN878 *Mtb* infection in transgenic FAT-ATTAC mice. (**A**) H&E-stained sections of uninfected and *Mtb*-infected RD-fed murine lung tissue showing infiltrated immune cells, fibrosis, lipid droplets and foamy macrophages, scale bar = 200 µm. Black arrow indicates bronchiole (lipid filled in infected), red arrow indicates epithelioid granuloma, and red * indicates fibrosis; (**B**) Histological grading of *Mtb*-infected lung pathology classified as immune cell infiltration, lipid droplet accumulation and foamy macrophages in males and females. Each class was graded on a 6-point scale ranging from 0 to 5 as discussed in the Methods section. The error bars represent the standard error of the mean. A Student’s *t*-test was conducted to calculate statistical significance. * *p* < 0.05, and ** *p* < 0.01 between indicated groups; and (**C**) Representative microphotographs of H&E-stained murine lung sections of *Mtb*-infected males and females fed on RD or MFD (fat gain) or FAB+ RD (fat loss). Black arrow indicates the presence of epithelioid and gigantocellular granulomas in 10× images. Red and green arrows indicate the presence of lipid droplets and foamy macrophages, respectively, and black * indicates fibrosis in the magnified 40× images.

To evaluate the impact of the loss or gain of fat on lung pathology, we conducted histological analyses. Hematoxylin & eosin (H&E)-stained sections of the lungs from infected RD-fed mice revealed significantly elevated levels of infiltrated immune cells, fibrosis, lipid droplets, and the presence of foamy macrophages compared to uninfected RD-fed mice (Figure 1A). Within the infected groups, for both males and females, we observed that levels of lipid droplets and foamy macrophages were higher in both FAB+ and MFD-fed mice, in comparison to the RD-fed group (Figure 1B,C). Histological sections of the lungs depicted the presence of epithelioid and gigantocellular granulomas, which were more or less confluent and lacked caseous necrosis (Figure 1C). These granulomatous lesions displayed a significant increase in lipid droplets and foamy macrophages in the lungs of mice subjected to FAB+ and those fed an MFD when compared to mice on an RD during the acute infection phase (Figure 1C, magnified 40× images). These findings indicate that both fat loss and fat gain can differentially regulate pulmonary pathology during acute *Mtb* infections in terms of infiltration of immune cells, foamy macrophages, and lipid accumulation. Our data primarily suggests that feeding on an MFD or undergoing FAB+ leads to increased lipid accumulation and foamy macrophages in the lungs of *Mtb*-infected mice, compared to RD-fed mice. Auramine Rhodamine staining of lung sections revealed highly colonized replicative *Mtb* in the lungs of FAB+ and MFD-fed mice (Supplementary Figure S3), indicating that increased fibrosis within the granulomatous lesions may contribute to the elevated *Mtb* replication.

### 2.3. Body Fat Alterations Induce Distinct Changes in Immune-Metabolic Signaling

**Figure 2:**
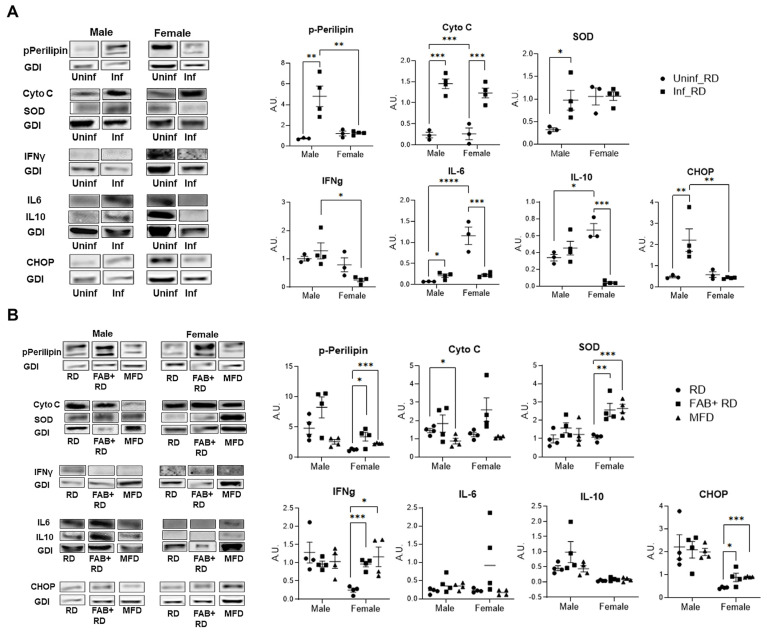
Body fat gain and fat loss induce alterations in lung lipid metabolism and inflammatory signaling between sexes during acute *Mtb*-infection. Immunoblot analysis of lipid hydrolysis marker (p-Perilipin), mitochondrial OX-PHOS markers (Cytochrome C and SOD), inflammatory markers (IFNγ, IL-6 and IL-10), and apoptosis marker (CHOP) in the lung protein lysates. Bar graph values were derived from densitometry analysis and by normalizing target protein expression to GDI. (**A**) Bar graphs illustrate the mean protein levels in both uninfected and infected RD-fed groups for males and females. (**B**) Bar graphs depict the variations in the mean protein levels of respective target protein among the different infected groups (RD-fed, FAB+ RD-fed and MFD-fed) for both males and females. The error bars represent standard error of the mean. Statistical significance was calculated using two-way ANOVA, with sex and infection as variables (**A**); and *t*-test, analyzing the changes with respect to RD groups in both male and female *Mtb*-infected mice (**B**). * *p* < 0.05, ** *p* < 0.01, *** *p* < 0.001 and **** *p* < 0.0001 between indicated groups. The complete immunoblot images for all the proteins are provided in the supporting data values file.

Our histological analysis revealed elevated levels of lipid droplets and fibrosis in the lungs of *Mtb*-infected mice (Figure 1A) [9,10]. Increased lipid accumulation can modify metabolic signaling in the lungs, subsequently influencing immune signaling and *Mtb* survival. To investigate whether the accumulated lipid droplets undergo hydrolysis and increase mitochondrial oxidative phosphorylation, we conducted immunoblotting analysis of the lung protein lysates (Refer to supporting data values file for complete immunoblot images). We probed for phospho-perilipin (a marker of lipid droplet hydrolysis), cytochrome C, and superoxide dismutase (SOD) (markers of mitochondrial oxidative phosphorylation) (Figure 2). Additionally, we analyzed whether increased hydrolysis of lipid droplets contributes to inflammatory signaling and cell death by measuring the protein levels of inflammatory markers (IFNγ, IL-6, and IL-10) and an apoptosis marker (Chop) by immunoblotting (Figure 2). First, we investigated whether there were any sex-related differences in the levels of these proteins during *Mtb* infection by comparing uninfected RD-fed and infected RD-fed male and female mice (Figure 2A). Our data revealed that in male infected mice, the levels of p-Perilipin (*p* = 0.0041), cytochrome C (*p* = 0.0001), SOD (*p* = 0.05), IL-6 (*p* = 0.05), and Chop (*p* = 0.0068) significantly increased in infected lungs, whereas in females, the levels of all these protein markers (except cytochrome C) either remained unchanged or significantly decreased compared to their respective sex-matched uninfected RD-fed mice (Figure 2A). Our data revealed that in female uninfected mice, the basal IL-6 level was significantly higher (*p* = 0.0001) compared to infected mice (Figure 2A). High levels of basal IL-6 (at the time of infection) may contribute to host defense in female mice through the stimulation of acute phase responses [19]. Overall, these data suggest a fundamental metabolic and immune difference in the lungs of RD-fed infected mice between males and females. Importantly, these data suggest that *Mtb* infection significantly increases lipid hydrolysis-induced apoptosis signaling associated with the pro-inflammatory cytokine IL-6 in the lungs of male mice.

Histological analysis demonstrated a further increase in the levels of lipid accumulation in the lung sections of the FAB+ and MFD-fed groups both in males and females (Figure 1C). The accumulated lipids may lead to lipotoxicity-induced cell death [20,21]. Next, we examined whether the levels of lipid droplet hydrolysis further increased in the lungs of infected FAB+ and MFD-fed mice (due to increased lipid accumulation in the lungs) compared to infected RD-fed mice by immunoblotting analysis (Figure 2B). Interestingly, we found that in male FAB+ and MFD-fed mice, the levels of lipid hydrolysis did not increase. However, in female FAB+ (*p* = 0.02) and MFD-fed mice (*p* = 0.0004), the levels significantly increased compared to sex-matched infected RD-fed mice. The levels of cytochrome C, SOD, IFNγ, IL-6, and IL-10 remained unchanged in male infected FAB+ and MFD-fed mice compared to male infected RD-fed mice (Figure 2B). However, in female infected FAB+ and MFD-fed mice, the levels of SOD and IFNγ significantly increased compared to female infected RD-fed mice (Figure 2B), indicating a robust antioxidant and IFNγ response in females within these groups, unlike the respective male groups. In addition, the levels of Chop significantly increased in female FAB+ (*p* = 0.05) and MFD-fed (*p* = 0.0015) mice (and were unaltered in males) compared to infected RD-fed mice (Figure 2B). These data revealed that both fat loss and fat gain significantly increase apoptosis and IFNγ signaling in females. Our data suggest that the accumulated lipids in the lungs, in the absence of sufficient lipid hydrolysis in fat loss and fat gain mice, likely have deleterious effects on infiltrated immune cells and may promote *Mtb* replication.

### 2.4. Sex-Specific Effects of Fat Loss and Fat Gain on Mtb Load in Mouse Lung Tissue

**Figure 3:**
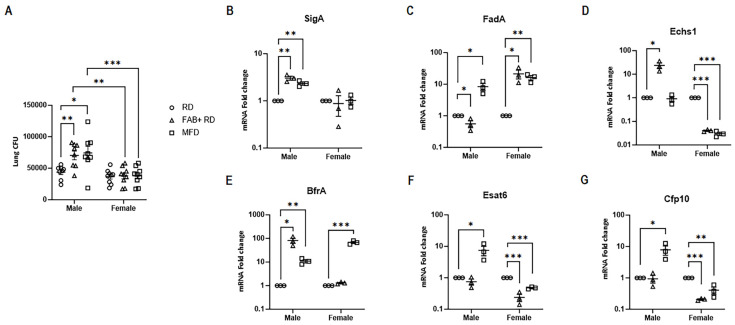
Loss and gain of body fat have distinct effects on pulmonary *Mtb* load and influence *Mtb* gene transcription differently between males and females. (**A**) CFU in lung homogenates of acutely infected FAT-ATTAC mice fed a regular diet (RD) and fat-ablated (FAB+ RD) or fed a medium fat diet (MFD). (**B**–**G**) Quantitative Real-Time PCR analysis of *Mtb*-specific genes (**B**) SigA, (**C**) FadA, (**D**) Echs1, (**E**) BfrA, (**F**) Esat6, and (**G**) Cfp10. The error bars represent standard error of the mean. Statistical significance was calculated using two-way ANOVA, with sex and alteration in body fat level as variables (**A**); and *t*-test, between two groups (compared to RD) in each sex (**B**–**G**). * *p* < 0.05, ** *p* < 0.01, and *** *p* < 0.001 between indicated groups.

We have demonstrated that fat loss and fat gain differentially impact pulmonary lipid metabolism, inflammatory signaling, and cell death in a sex-specific manner during acute *Mtb* infection (Figure 2). Such sex-specific disparities can lead to differential pulmonary bacterial burden. Therefore, we analyzed whether fat loss and fat gain can influence *Mtb* load in the lungs and whether this differs between infected male and female mice. We did not observe any significant difference in *Mtb* load between males and females in RD-fed mice. Significant differences were observed in *Mtb* lung colony-forming units (CFU) between male and female infected mice subjected to fat loss or fat gain (FAB+ RD, 95% Confidence Interval (CI) 1.13 to 5.38, *p* = 0.0013, MFD, 95% Confidence Interval (CI) 1.51 to 5.7, *p* = 0.0003) (Figure 3A). In males, both fat loss and fat gain significantly increased (FAB+ RD, *p* = 0.0015, MFD, *p* = 0.042) the *Mtb* load in the lungs, while in females, no significant change was observed. This data supports our notion that impaired lipid hydrolysis in the fat loss and fat gain groups of infected males may have contributed to the increased *Mtb* levels in these mice.

### 2.5. Impact of Body Fat Alterations on Mtb Gene Expression

Our data indicated variations in lung bacterial burden between males and females, along with differences in lung metabolic and immune signaling during *Mtb* infection in both sexes when subjected to fat loss or fat gain. This indicates that the altered metabolic state of the lungs may regulate metabolic genes in *Mtb*, influencing its dormant or replicative status, which, in turn, could impact the lung bacterial burden. The lung pathology depends on the level of replication and persistence of *Mtb* and the host immune system. To investigate the possible correlation between lung bacterial burden and the metabolic status in the lungs, we conducted an analysis of certain *Mtb*-specific genes using qPCR. First, we examined the levels of the *Mtb*-SigA gene in the lungs of infected mice (Figure 3B). We found that the levels of SigA significantly increased in FAB+ (*p* = 0.002) and MFD-fed (*p* = 0.002) groups in male mice but remained unchanged in female mice compared to their respective sex-matched infected RD-fed groups (Figure 3B). These data align with the lung bacterial burden (Figure 3A), suggesting that fat gain and fat loss have a lesser impact on lung *Mtb* load in females, whereas they promote *Mtb* load in the lungs of males.

We examined the impact of fat loss and fat gain on *Mtb*’s fatty acid metabolism by analyzing the expression levels of genes involved in this process, specifically FadA (Fatty-acid degradation A) and Echs1 (Rv3774, Enoyl-CoA hydratase) (Figure 3C,D). FadA functions as an acetyltransferase, converting host acetyl-CoA to acetoacetyl-CoA, while Echs1 catalyzes the second step in the crucial beta-oxidation pathway of fatty acid metabolism. MFD increased the levels of FadA significantly in pulmonary *Mtb* in both males (*p* = 0.02) and females (*p* = 0.004), whereas FAB+ significantly increased the levels of Fad A only in female (*p* = 0.03) mice compared to their sex-matched infected RD-fed mice. However, the levels of Echs1 significantly increased (*p* = 0.02) in FAB+ and remained unaltered in MFD-fed male groups, and they significantly decreased in both FAB+ and MFD-fed female groups compared to their sex-matched RD-fed groups (Figure 3D). These data suggest that the assimilation and conversion of host acetyl-CoA, as well as the level of fatty acid oxidation in *Mtb* within the lungs, depend on the host microenvironment, which differs between males and females and can be further influenced by body fat loss and fat gain. Changes in intracellular fatty acid degradation and beta-oxidation could lead to oxidative stress. Next, we assessed the levels of the iron-homeostasis maintenance gene BfrA (bacterioferritin), which plays a significant role in protecting *Mtb* from oxidative stress (Figure 3E). qPCR analysis revealed that the levels of BfrA significantly increased in both FAB+ (*p* = 0.01) and MFD-fed (*p* = 0.005) groups in males and only in the MFD-fed group in females (*p* = 0.0005) compared to their respective sex-matched infected RD-fed groups (Figure 3E).

Subsequently, we assessed the replication status of *Mtb* by analyzing the expression of the Esat6 and Cfp10 genes (Figure 3F,G). In males, the levels of both Esat6 and Cfp10 significantly increased or remained unaltered in both FAB+ and MFD-fed groups compared to RD-fed mice. In contrast, in females, both Esat6 and Cfp10 significantly decreased compared to RD-fed mice (Figure 3F). These data suggest the following: (i) fat loss and fat gain have a specific impact on *Mtb* replication in males, and (ii) *Mtb* may utilize the accumulated lipids in the lungs for survival. The increased mitochondrial oxidation in males likely contributes to their replication status in the lungs.

### 2.6. Effect of Body Fat Alterations on Immune Cell Population and Their Activation Status

**Figure 4:**
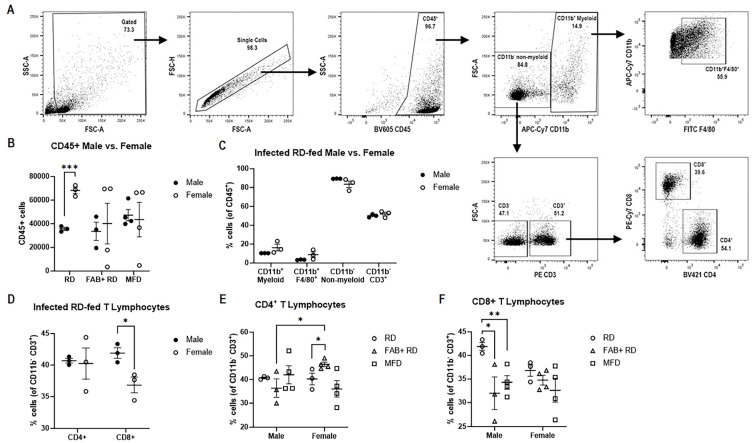
Flow cytometry analysis of *Mtb*-infected murine lung. (**A**) Gating strategy for immunophenotyping infiltrated immune cell population; (**B**) Bar graph showing the frequencies of CD45^+^ cells between male and female and among different diet and FAB+ groups; (**C**) Bar graph showing the percentage of CD11b^+^ myeloid, CD11b^+^F4/80^+^ (double positive), CD11b^−^ non-myeloid and CD11b^−^CD3^+^ cells from parent CD45^+^ population in infected RD-fed male and female mice; (**D**) Bar graph showing the percentage of CD4^+^ and CD8^+^ cells from parent CD11b^−^CD3^+^ T-lymphocytes between infected RD-fed males and females; and (**E**,**F**) Bar graph showing the percentage of CD4^+^ and CD8^+^ T-cells among different diet and FAB+ groups in males and females. The error bars represent the standard error of the mean. Statistical significance was calculated using a *t*-test between male and female (**B**–**D**); and two-way ANOVA, with sex and alteration in body fat level as variables (**E**,**F**). * *p* < 0.05, ** *p* < 0.01, and *** *p* < 0.001 between indicated groups.

CD11b^+^ myeloid cells, as well as lymphoid cells such as T-cells and NK cells, play crucial roles in defending against pulmonary *Mtb* infection and the formation of granulomatous lesions [22,23]. To assess the population of infiltrated immune cells in the infected lungs among different groups, including those on stimulated fat loss and induced fat gain, and between males and females, we conducted immunophenotyping analysis in the infected lungs using flow cytometry (Figure 4A). The number of total CD45^+^ leukocytes was significantly higher in the lungs of infected female mice (*p* = 0.0004) compared to infected male mice between RD-fed groups (Figure 4B), indicating that females had a higher influx of leukocytes into their lungs compared to males during *Mtb* infection, which aligns with our histological observations. The total CD45^+^ leukocyte numbers remained unchanged in all the infected groups (with fat loss or fat gain) compared to the infected RD-fed group in both males and females (Figure 4B).

Among the different CD45^+^ leukocytes, we then determined the proportions of various myeloid and lymphoid leukocyte populations. Our findings revealed that the proportions of CD11b^+^ myeloid, CD11b^+^F4/80^+^ macrophages, CD11b^−^ non-myeloid, and CD11b^−^CD3^+^ T-cells (expressed as a percentage of CD45^+^ cells set to 100%) showed no significant differences between infected males and females in the RD-fed group (Figure 4C). Subsequent analysis of the infected groups demonstrated that, in males, fat loss increases CD11b^+^ myeloid cells and decreases CD11b^−^ non-myeloid cells (Supplementary Figure S4A). Whereas, in females both fat loss and fat gain have no significant effect on the levels of CD11b^+^ myeloid, CD11b^+^F4/80^+^ macrophages, CD11b^−^ non-myeloid, and CD11b^−^CD3^+^ T-cells (Supplementary Figure S4B). 

Next, we conducted an analysis to determine if, within the CD11b^−^CD3^+^ T-cell population, the CD4^+^ and CD8^+^ cell populations (expressed as a percentage of CD11b^−^CD3^+^ cells set to 100%) exhibit any differences between infected RD-fed groups of males and females. We found that there was no significant difference in the CD4^+^ T-cells, but the CD8^+^ T-cells were substantially higher in males (*p* ≤ 0.05) compared to females in the RD-fed groups (Figure 4D). Further analysis of the various infected groups with fat loss or fat gain revealed that in males, there were no significant differences in the levels of CD4^+^ cells when compared to the RD-fed infected group (Figure 4E). However, in females, fat loss in infected mice significantly increased the levels of CD4^+^ cells compared to the RD-fed infected group (Figure 4E). Notably, our data also demonstrated that the female FAB+ group had significantly higher levels of CD4^+^ cells (*p* = 0.03) compared to the male FAB+ group (Figure 4E). Importantly, both fat loss (*p* = 0.01) and fat gain (*p* = 0.03) significantly reduce the levels of CD8^+^ cells in males and remained unchanged in females compared to their sex-matched RD-fed groups (Figure 4F). These findings indicate how fat loss and fat gain can finely modulate the levels of various immune cell populations differently in males and females during *Mtb* infections. Overall, this suggests that: (i) RD-fed males exhibit higher levels of CD8^+^ cells in the lungs compared to females, and (ii) fat loss and fat gain significantly reduce CD8^+^ cells in the lungs in males during infection.

To gain additional preliminary insights into the CD4^+^ and CD8^+^ T-cell subsets, based on the expression levels of intracellular phenotypic markers, we adopted a sample pooling approach (n = 4) based on their respective infected groups and sex, as described in the methods section. This approach was necessary due to the technical challenges associated with the simultaneous isolation of these cells and the execution of experimental procedures, such as T-cell stimulation and flow cytometric analysis (from a total of 32 samples). We processed the samples in two separate batches (Supplementary Figure S5). Initially, we isolated TCRβ^+^CD4^+^ and TCRβ^+^CD8^+^ cells through surface staining. The percentages, calculated as proportions of 100% TCRβ^+^ cells, revealed that CD8^+^ cells were more abundant in infected male RD-fed mice compared to infected female RD-fed mice, while CD4^+^ cells were notably not significantly different between the sexes in infected RD-fed mice. Despite the higher initial levels of CD8^+^ cells in the male RD-fed group as compared to the female RD-fed group, the levels of CD8^+^ cells decreased in male mice with stimulated fat loss and induced fat gain groups, in comparison to the male RD-fed group.

To evaluate the cellular subtypes of the lung-infiltrating TCRβ^+^CD4^+^ and TCRβ^+^CD8^+^ T-cells, we analyzed the pooled single-cell suspensions for evaluating the expression of cytokines and transcription factors through intracellular staining. These cytokines included IFNγ, TNFα, and IL-2, and transcription factors FOXP3, IL-4, and IL-17, following stimulation with PMA and ionomycin in combination with protein transport inhibitors. Our data revealed distinct phenotype patterns among the groups and between sexes (Table 1). For instance, in males, fat loss and fat gain showed a trend towards increased levels of IFNγ and decreased levels of TNFα in stimulated CD4^+^ cells compared to RD-fed mice. This suggests a higher cytotoxic response in lung CD4^+^ T-cells in RD-fed mice compared to other groups, indicating an increased Th1 response with fat loss or fat gain. In females, fat loss decreased both IFNγ and TNFα levels in stimulated CD4^+^ cells but increased FOXP3 and IL-4 levels compared to RD-fed mice (Table 1). This suggests that fat loss in females decreased the cytotoxic response in lung CD4^+^ T-cells and increased Treg and Th2 responses (based on the levels of FOXP3 and IL4 expression, respectively) compared to RD-fed mice (Table 1). Interestingly, induced fat gain via feeding an MFD significantly reduced IFNγ and TNFα levels in stimulated CD8^+^ cells but increased FOXP3 levels compared to RD-fed mice in both males and females (Table 1), indicating that fat gain may increase the Treg population during *Mtb* infection. Overall, our data indicate that fat loss increases Treg cells in CD4^+^ cells, and fat gain increases Treg cells in CD8^+^ cells in both male and female infected mice fed an RD.

In summary, our flow cytometry-based immunophenotyping analysis indicated that the female RD-fed group has significantly higher levels of CD45^+^ cells compared to the male RD-fed group, suggesting a greater immune cell infiltration in the lungs of females during *Mtb* infection. However, the significantly increased CD8^+^ T-cell levels in the male RD-fed group compared to the female RD-fed group suggest a stronger cytotoxic effect in the lungs of males compared to females. This observation is also supported by the data showing increased levels of TNFα and IL-2 expressing CD8^+^ T-cells in males. In addition, our data indicate that fat loss increases Treg CD4^+^ cells, and fat gain increases Treg CD8^+^ cells in both male and female infected mice fed an RD.

### 2.7. Sex-Specific Differences in T-Cell Distribution in Granulomatous Lesions Based on Body Fat

**Figure 5:**
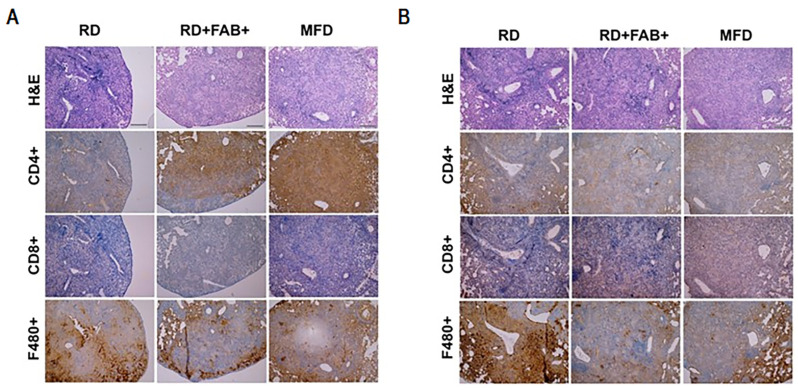
Fat loss and fat gain influence the distribution of immune cells within granulomatous lesions in a sex-specific manner. Representative immunohistochemistry (IHC) images of the *Mtb*-infected murine lung sections stained with CD4, CD8, and/or F4/80 antibodies in males (**A**) and females (**B**). Magnification-4×; Scale bar = 100 µm. Lung sections were from RD-fed, FAB+ RD-fed, and MFD-fed groups.

As flow cytometry does not allow a direct assessment of the spatial distribution of the leukocytes within the granuloma, we also investigated whether fat gain and fat loss influence the population of immune cells within the granulomatous lesions and whether these differences vary by sex in *Mtb* infection. Immunohistochemistry analysis of CD4, and CD8 in lung sections revealed significant differences in the distribution of these immune cells within the granulomas among different infected groups and sexes. In males, both fat loss and fat gain increased the levels of CD4 within the lung granulomas compared to RD-fed mice (Figure 5A). In contrast, in females, alterations in body fat did not change the levels of CD4 within the granulomas compared to RD-fed mice (Figure 5B). There was no significant change in the levels of CD8 observed between the groups in both males and females. These findings indicate that, in the context of *Mtb* infection, fat loss and fat gain differentially affect the distribution of T-cells within granulomatous lesions, in a sex-specific manner. Overall, our immunophenotyping analyses using flow cytometry and IHC uncovered variations in both the overall levels of T-cell infiltration in the lungs and the spatial distribution of T-cells within granulomas across different sexes and in relation to body fat gain or fat ablation.

### 2.8. Mtb Infection Reduces Pro-Inflammatory Status of CD8^+^ Cells in Males during Fat Level Alterations

**Figure 6:**
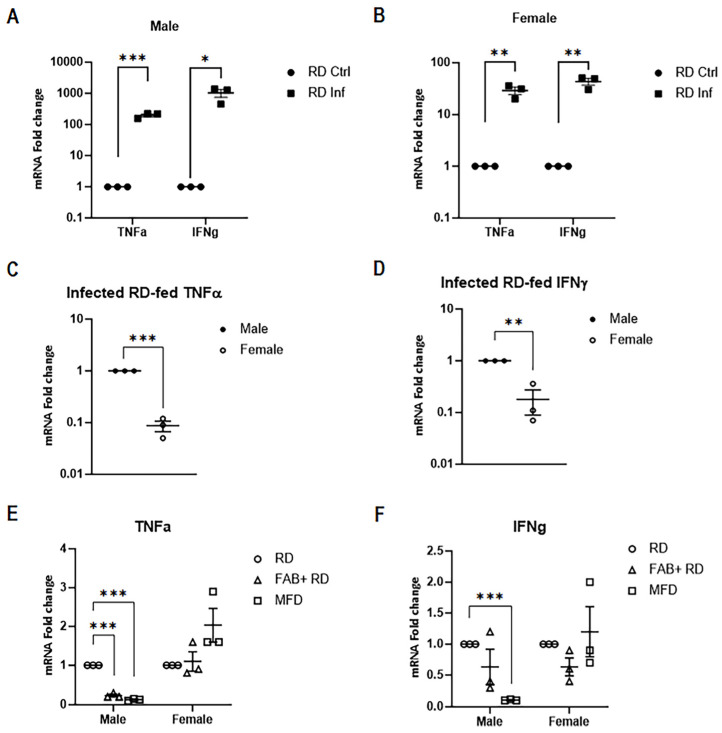
Male CD8^+^ cells showcase greater pro-inflammatory potential in RD-fed mice during acute *Mtb* infection compared to females as demonstrated by qPCR analysis. Fold change increase in TNFα and IFNγ mRNA transcripts in naïve CD8^+^ T-cells in uninfected RD-fed (RD Ctrl) mice compared to *Mtb*-infected RD-fed (RD Inf) mice in males (**A**) and in females (**B**). Fold change in TNFα (**C**) and IFNγ (**D**) compared between infected RD-fed males and females. Fold change in TNFα (**E**) and IFNγ (**F**) between the different groups (RD, FAB+ RD and MFD) compared to infected RD-fed mice in males and females. The error bars represent the standard error of the mean. Statistical significance was calculated using a *t*-test. * *p* < 0.05, ** *p* < 0.01, and *** *p* < 0.001 between indicated groups.

CD8^+^ T-cells have been identified as a critical component of granulomas found in the lung tissue of mice with tuberculosis, playing a pivotal role in host resistance against the disease. Specifically, the release of IFNγ and TNFα by CD8^+^ T-cells acts synergistically to activate macrophages, thereby enhancing their microbicidal activity [24,25]. These activated T-cells are responsible for reducing the bacterial load as well as increasing inflammatory damage in the lungs [26]. We analyzed the mRNA levels of IFNγ and TNFα in resting (unstimulated) CD8^+^ T-cells isolated from the lungs of infected mice and from the spleens of uninfected male and female mice. qPCR analysis demonstrated significantly increased TNFα and IFNγ levels in CD8^+^ cells in infected RD-fed mice compared to uninfected RD-fed mice in both males and females (Figure 6A,B). However, the upregulation levels of TNFα (*p* = 0.0001) and IFNγ (*p* = 0.0001) were significantly higher in infected males fed an RD compared to infected females fed an RD (Figure 6C,D). This suggests that CD8^+^ T-cells derived from male infected RD-fed mice are more pro-inflammatory than CD8^+^ T-cells derived from female infected RD-fed mice, which is also evident from the flow cytometric analysis (Table 1).

To investigate whether fat loss or fat gain have differential effects on pro-inflammatory gene expression in CD8^+^ T-cells, we examined the mRNA transcript expression levels of TNFα and IFNγ in resting CD8^+^ T-cells isolated from different infected groups (FAB+ and MFD-fed), and compared their levels to those in the infected RD-fed group, separately for male and female mice (Figure 6E,F). qPCR analysis revealed that fat loss significantly reduced the levels of IFNγ, whereas fat gain significantly decreased the levels of both TNFα and IFNγ in CD8^+^ cells compared to RD-fed mice in males. Conversely, there were no significant changes in the levels of TNFα and IFNγ between the infected female groups with fat loss or fat gain (Figure 6E,F). These data indicate that body fat levels significantly influence the expression levels of pro-inflammatory genes in CD8^+^ T-cells in males but not in females during infection.

### 2.9. Fat Loss and Fat Gain Perturb Mitochondrial Gene Expression in CD8^+^ T-Cells in a Sex-Dependent Manner during Mtb Infection

**Figure 7:**
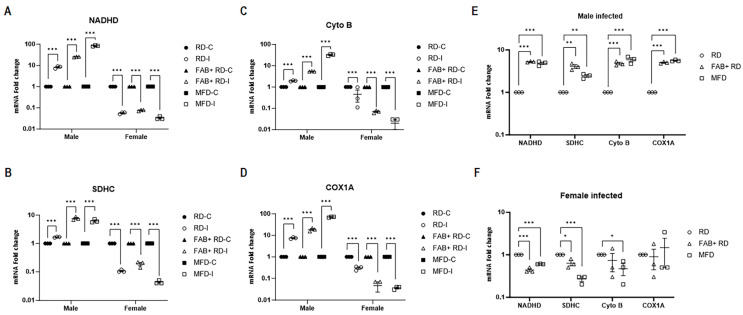
Acute *Mtb* infection triggers mitochondrial oxidative phosphorylation in lung CD8^+^ T cells in males, in contrast to females. Quantitative Real-Time PCR analysis of mitochondrial genes Nadhd (**A**), Sdhc (**B**), Cyto B (**C**), and Cox1A (**D**) in naïve CD8^+^ T-cells between uninfected and *Mtb*-infected male and female mice on RD, FAB+ RD and MFD. Bar graph showing the mRNA expression levels compared between the groups of *Mtb*-infected males (**E**) and females (**F**), separately. The error bars represent the standard error of the mean. Statistical significance was calculated using a *t*-test. * *p* < 0.05, ** *p* < 0.01, and *** *p* < 0.001 between indicated groups.

Mounting evidence suggests that mitochondrial dysfunction can polarize innate immune responses and increase host susceptibility to certain pathogens by altering mitochondrial metabolism and homeostasis [27,28]. Given the increased accumulation of lipid droplets in the lungs and the altered lung metabolic status in *Mtb*-infected mice, it is critical to examine whether the increased lipid droplets in the lungs affect mitochondrial oxidative phosphorylation in lung-infiltrated CD8^+^ T-cells. Therefore, we examined the mRNA expression levels of NADH (NADH dehydrogenase, Complex 1), SDHC (Succinate dehydrogenase, c subunit of Complex 2), Cyto B (Cytochrome bc1, Complex 3), and COX1A in the mitochondrial respiratory chain of unstimulated CD8^+^ T-cells isolated from the lungs of *Mtb*-infected mice by qPCR analysis. We found that the mRNA levels of all the tested mitochondrial genes were significantly upregulated in CD8^+^ cells in male-infected RD-fed mice, infected fat ablated mice and infected fat induced mice (*p* = 0.0003) and significantly downregulated in female-infected RD-fed mice, infected fat ablated mice and infected fat induced mice (*p* = 0.0004) compared to their respective sex-matched uninfected mice (Figure 7A–D). However, the levels of all the tested mitochondrial genes were either significantly upregulated or remained unaltered in infected female RD-fed mice compared to infected male RD-fed mice (Supplementary Figure S6). These data suggest that the basal levels of mitochondrial oxidative phosphorylation in resting CD8^+^ cells are significantly higher in infected females compared to infected males. These data also suggest that the increased mitochondrial oxidative phosphorylation in female RD-fed mice might have led to decreased expression levels of pro-inflammatory cytokines (TNFα and IFNγ) in females compared to male RD-fed mice during *Mtb* infection.

Next, we examined the effect of fat loss and fat gain on the levels of mitochondrial genes in CD8^+^ cells in infected males and females (Figure 7E,F). In infected males, both fat loss and fat gain either significantly increased the levels of mitochondrial genes in CD8^+^ cells or left them unaltered compared to RD-fed mice. However, in infected females, both fat loss and fat gain significantly reduced the levels of these genes in CD8^+^ cells or left them unaltered compared to RD-fed mice. Our data indicate a significant increase in the levels of mitochondrial oxidative phosphorylation genes in lung CD8^+^ T-cells in RD-fed mice with either fat loss or fat gain, which might have led to a decreased expression of TNFα and IFNγ in male mice, while the opposite is true in female mice. Overall, our data suggest that body fat loss or fat gain differentially regulate oxidative phosphorylation signaling in CD8^+^ cells and thus can influence the activation and functioning of CD8^+^ cells in a sex-biased manner during *Mtb* infection.

### 2.10. Females Exhibit Strong Systemic IFNγ Response, Irrespective of Body Fat Alterations

**Figure 8:**
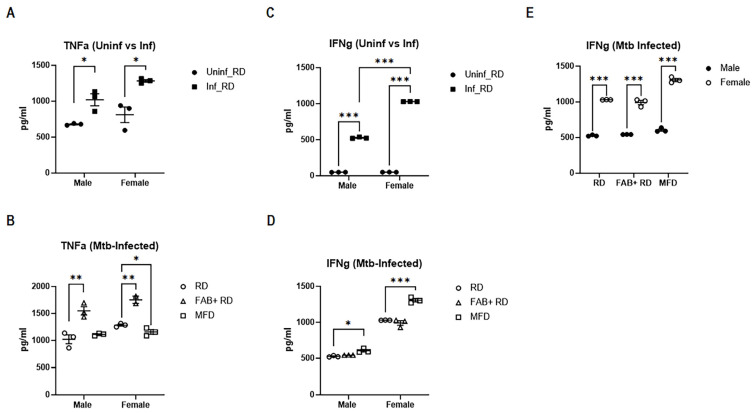
Females exhibit stronger systemic IFNγ response against *Mtb* infection. Representative bar graphs showing circulatory cytokine levels of TNFα (**A**,**B**) and IFNγ (**C**–**E**) in the plasma of uninfected and *Mtb*-infected male and female mice as analyzed by ELISA. The error bars represent the standard error of the mean. Statistical significance was calculated using a *t*-test. * *p* < 0.05, ** *p* < 0.01, and *** *p* < 0.001 between indicated groups.

Previously, we have shown that *Mtb* infection can selectively affect the levels of inflammatory cytokines in the lungs and adipose tissue during acute infection, which can subsequently influence the overall systemic cytokine profile [29]. Since the protective immune response against *Mtb* relies significantly on cytokine production, we conducted an analysis of systemic inflammatory cytokine levels in *Mtb*-infected mice, taking into account their fat loss and fat gain statuses. We measured the circulating levels of pro-inflammatory cytokines, such as TNFα and IFNγ, in mouse plasma by ELISA (Figure 8A–D). The plasma levels of TNFα significantly increased in infected RD-fed mice compared to uninfected RD-fed mice in both males and females (Figure 8A). We further assessed whether fat loss or fat gain differentially regulate systemic levels of TNFα and IFNγ in males and females. Our data indicated that fat loss significantly increases TNFα levels and does not alter IFNγ levels in both males and females compared to RD-fed mice during infection (Figure 8B,D). Whereas fat gain decreases TNFα only in females but significantly increases IFNγ in both males and females compared to RD-fed mice during *Mtb* infection (Figure 8B,D). The plasma levels of IFNγ also significantly increased in infected RD-fed mice compared to uninfected RD-fed mice in both males and females (Figure 8C). Notably, we observed an elevated IFNγ response in female mice in all groups compared to their respective male counterparts during infection (Figure 8E). These data suggest that females may mount a stronger systemic IFNγ response against *Mtb* infection, potentially contributing to reduced extra-pulmonary *Mtb* infection compared to male mice, which needs to be examined. However, unlike the systemic levels, in female mice, we observed a significant reduction in IFNγ levels in the lungs in infected RD-fed mice compared to uninfected RD-fed mice, suggesting that the localized and systemic inflammatory responses to *Mtb* infection may differ.

Thus, our overall data indicate significant differences in cytokine levels and proinflammatory signaling at the systemic (circulatory), localized (lung tissue), and immune cell (CD8^+^ cells) levels, and these differences are observed between sexes during *Mtb* infection. Our data demonstrated that, at the systemic level, females exhibit a strong IFNγ response, whereas at the localized level, males display a robust IL-6 response. Although CD8^+^ cells in the lungs of infected mice express higher levels of IFNγ and TNFα compared to uninfected mice, the mRNA levels of the proinflammatory cytokines were significantly higher in RD-fed males compared to females.

## 3. Discussion

The WHO reports a significantly higher risk of TB contraction and mortality among men worldwide compared to women [4]. However, the factors contributing to this increased susceptibility of men to tuberculosis remain incompletely understood. It’s crucial to acknowledge sex differences in body fat levels and distribution [30,31], with diet playing a pivotal role in regulating body fat [32]. Our previous research has shown that both acute fat loss induced by fat cell apoptosis and obesity resulting from a high-fat diet led to increased pulmonary *Mtb* burden and pathology in murine models infected with H37Rv [9,10]. However, the mechanisms by which changes in body fat levels impact the activation of systemic, localized, and pulmonary immune cells, as well as their influence on *Mtb* survival, replication, dormancy, metabolism, and pulmonary *Mtb* burden during acute *Mtb* infection, have not been explored. Furthermore, our previous research did not investigate whether changes in body fat levels, whether fat loss or fat gain, elicit varying immune responses to *Mtb* infection in males and females. Our current study represents a pioneering investigation into several aspects, notably the impact of body fat loss or gain on gene regulation in lung-infiltrated CD8^+^ cells, with particular emphasis on sex-dependent differences. This exploration has yielded significant insights. Firstly, we observed that *Mtb* infection triggers pronounced pulmonary pathology in males, despite no significant difference in pulmonary *Mtb* load compared to females. This outcome is attributed to shifts in metabolic signaling, characterized by heightened lipid hydrolysis, and an increase in proinflammatory signaling driven by IL-6 and localized pro-inflammatory CD8^+^ cells. These factors could contribute to apoptotic cell death in males compared to females fed an RD. Secondly, we found that in males, fat loss or fat gain increases the *Mtb* burden in the lungs compared to females during acute *Mtb* infection, which is attributed to impaired lipid metabolism in the lungs and increased mitochondrial oxidative phosphorylation-regulated activity in CD8^+^ cells in the lungs during *Mtb* infection. Furthermore, we have demonstrated that females exhibit a stronger systemic IFNγ response compared to males during *Mtb* infection, which may either prevent active disease or contribute to latency in females during *Mtb* infection [33].

TB is the most prevalent bacterial infectious disease in humans and a leading cause of death, with a higher mortality rate in men compared to women. The underpinning factors behind this apparent sex disparity are often attributed to societal and cultural roles, behaviors, and sex-related aspects. Previous studies in murine models, including H37Rv (a laboratory-adapted *Mtb* strain) and HN878 (a hypervirulent clinical strain), have revealed that impaired formation of B cell follicles and reduced levels of IL-23 in the lungs of male mice increase susceptibility to *Mtb* infection in males [34]. A robust inflammatory response in the lungs of *Mtb*-infected males has been identified as a causative factor in pulmonary damage, contributing to sex inequality in *Mtb* infection [14]. Our study further demonstrates that males infected with *Mtb* exhibit a more robust proinflammatory response in their lung tissue, characterized by elevated levels of IL-6 and TNFα in their CD8^+^ T-cells, compared to females. This heightened response likely contributes to increased cell death, as evidenced by elevated Chop levels in the lungs of *Mtb*-infected males. This increased apoptosis is associated with tissue damage, which may play a role in the development of pulmonary pathology in *Mtb*-infected males [35].

Our study also revealed that lipid metabolism in the lungs plays a pivotal role in determining the inflammatory status of the lungs. Overall, immunoblotting data revealed significant variations between the groups (with fat loss and fat gain) and between males and females, suggesting that lipid hydrolysis levels (p-Perilipin) and apoptotic cell death (CHOP) are significantly higher in infected male mice compared to infected female mice fed an RD. Cellular lipid metabolism is intricately linked to mitochondrial oxidative phosphorylation [36], which plays a crucial role in the activation of immune cells [37,38]. Understanding immunometabolism and its associated bioenergetic pathways is essential for unraveling the intricate connections between metabolic status and the functional roles of immune cells [37,38]. Additionally, compromised metabolic reprogramming within CD8^+^ T-cells may be associated with their dysfunction during *Mtb* infection [39]. It is well known that the generation of adenosine triphosphate (ATP) in naive T-cells depends on oxidative phosphorylation (OXPHOS), and activated T-cells reprogram their metabolism toward aerobic glycolysis to produce ATP [40]. Our data suggest that increased mitochondrial oxidation genes in female mice might have resulted in reduced levels of pro-inflammatory signaling in resting CD8^+^ cells, contrasting with male mice fed an RD during *Mtb* infection (Supplementary Figure S6).

Our current study has shown that the acute loss of body fat and gain of body fat increase the severity of pulmonary *Mtb* infection in males compared to females, orchestrated by various mechanisms at the systemic, localized (lungs), and cellular (lung CD8^+^ T cell) levels. Infection with *Mtb* triggers a robust localized inflammatory reaction, which plays a crucial role in the development of tuberculosis. *Mtb* activates components of the innate immune response, including the recruitment of polymorphonuclear (PMN) and mononuclear phagocytes, as well as the induction of pro-inflammatory cytokines like tumor necrosis factor α (TNFα). These responses occur early after *Mtb* infection but can persist as the organism establishes itself within granulomas [41]. Previously, we have shown that *Mtb* infection can selectively affect the levels of inflammatory cytokines in the lungs and adipose tissue during acute infection, which can subsequently influence the overall systemic cytokine profile [29]. In our present study, we observed that the systemic IFNγ response is less robust in males compared to females. At the local level, baseline IL-6 levels were notably higher in the lungs of uninfected females compared to males (data not presented), potentially enhancing the early immune response in females and reducing the *Mtb* burden during the initial stages of infection [42]. During infection, while IL-6 levels increase in the lungs of infected RD-fed male mice (compared to uninfected mice), and IL-6 levels do not decrease in mice with either fat loss or fat gain, the elevated *Mtb* burden in males with fat loss and fat gain may be attributed to changes in other cellular signaling, such as metabolic signaling. This could potentially contribute to the increased *Mtb* burden in the lungs [10,43]. Our data revealed that the alteration in metabolic signaling is likely a result of impaired lipid hydrolysis in the lungs. This leads to the accumulation of lipids, which can facilitate the replication of *Mtb* by inducing the upregulation of genes related to fatty acid beta-oxidation, as well as replication genes such as Esat6 and Cfp10 [43,44]. At the cellular level in the infected lungs, the levels of CD8^+^ cells were consistently higher in males compared to females, regardless of their body fat levels. Interestingly, fat loss and fat gain exhibited contrasting effects on CD8^+^ cells in male and female infected mice, leading to significantly decreased mRNA levels of pro-inflammatory TNFα and IFNγ in male CD8^+^ cells. This could be attributed to the increased mitochondrial oxidative phosphorylation in CD8^+^ cells in males with fat loss or fat gain.

Our study was designed to investigate the effects of metabolic conditions, such as induced fat loss or fat gain, on the differences in pulmonary pathology and *Mtb* burden in male and female mice. Previously, we and others have shown that *Mtb* infects and persists in adipose tissue [9,10,29,45,46,47]. *Mtb* infection alters adipose tissue physiology, resulting in a loss of adipocytes [10,45]. An acute loss of body fat is a detrimental factor in active TB disease [9]. A retrospective cohort study concluded that patients losing weight during TB treatment, especially in the first month, should be closely monitored as they are at higher risk of treatment failure or death [48]. These factors indicate that adipose tissue and body fat likely play a major role in regulating pulmonary pathogenesis and the activation/reactivation of TB. Adipose tissue, rich in adipocytes, plays a crucial role in regulating whole-body immune and metabolic homeostasis [49,50]. Adipocyte-derived adipokines, such as adiponectin and leptin, regulate immune and metabolic functions in immune cells [51,52,53]. Decreased leptin levels have been shown to be associated with wasting and inflammation in TB patients [54]. Acute loss of adipocytes or body fat causes dyslipidemia and leads to lipid accumulation in various organs [55], and the accumulated lipid droplets can inhibit the phagocytic activation of macrophages [56,57]. In our previous study using the H37Rv (lab-adapted strain) infected FAT-ATTAC murine model, we demonstrated that FAB+ leads to increased lipid accumulation in the lungs and induces M2 polarization [9]. The absence of M1 macrophages in the lungs fails to clear *Mtb* and increases CFU [58]. In our earlier study, we could not demonstrate a significant difference in CFU between FAB+ and FAB− mice since the study was not conducted separately in males and females. However, in the current study, we demonstrated that FAB+ (fat loss) significantly increases lung CFU in males but not in females, suggesting that the loss of body fat is more detrimental in males during *Mtb* infection.

Studies have shown that overweight individuals (not diabetic or obese) are less susceptible to TB [59]. In our study, feeding a medium-fat diet (MFD) mimicked an overweight condition in mice, and our data indicated that feeding an MFD does not protect male mice from *Mtb* infection. In fact, it increased the expression of replication-related genes in *Mtb* (ESAT6 and CFP10) and caused a significant increase in lung CFU in male mice compared to mice fed a regular diet (RD). This may be because our study primarily focuses on acute infection. However, clinical data demonstrating reduced susceptibility to TB in individuals with moderately higher BMI (those who are not diabetic or obese) do not pertain solely to acute conditions. The limitation of our study is that we have examined the impact of fat loss or fat gain on the regulation of lung metabolic and immune signaling, as well as *Mtb* burden, only during acute infection. Consequently, further investigations are required to gain a comprehensive understanding of the role of body fat in chronic TB infection.

Our research underscores the significant impact of fluctuations in body fat on lung pathology, spanning metabolic, inflammatory, apoptotic signaling, gene regulation, and *Mtb* replication within the lungs. It is evident that body fat levels and metabolic status play a pivotal role in determining pulmonary pathology and *Mtb* load, particularly in male mice. In *Mtb*-infected males fed an RD, a robust CD8^+^ T-cell response, coupled with proinflammatory signaling, can result in inflammatory damage and cell death in the lungs. In contrast, infected females exhibit a robust systemic IFNγ response and reduced lung CD8 levels, suggesting a tendency towards latency. Overall, our data indicate that fat gain and fat loss differentially regulate processes such as lipid hydrolysis, mitochondrial oxidative phosphorylation, oxidative stress, cell death, as well as immune cell infiltration and activation in the lungs, with varying effects on pulmonary pathology and *Mtb* load in both males and females during acute *Mtb* infection. Importantly, these effects are more pronounced in males during *Mtb* infection.

## 4. Materials and Methods

### 4.1. Ethics Statement

All animal experimental procedures received approval from the Institutional Animal Care and Use Committee (IACUC) and Institutional Biosafety Committees of the Center for Discovery and Innovation (CDI) at Hackensack University Medical Center and were conducted in accordance with the guidelines set forth by the National Research Council.

### 4.2. Mtb Infection and Animal Model

The transgenic FAT-ATTAC mice, both male and female and on a C57BL/6J background, were bred (from the same litters) and housed at the CDI Research Animal Facility (RAF) in groups of 4–5 animals per sterilized filter-top cage. They were maintained under a 12-h light-dark cycle, with controlled humidity and temperature conditions. The number of mice used per group in the study were based on our previous studies [9,10,29]. At the age of 4–5 weeks, we obtained a total of 48 male mice and 51 female mice, which were then randomly assigned to two groups: RD and MFD. The RD groups consisted of male (n = 32) and female (n = 34) mice, which were fed a regular control diet (RD: 10 kcal% fat, 20 kcal% protein, and 70 kcal% carbohydrate) (#D12450J, Research Diets, Inc., New Brunswick, NJ, USA). The MFD groups comprised male (n = 16) and female (n = 17) mice, which were fed a medium-fat diet (MFD: 30 kcal% fat, 20 kcal% protein, and 50 kcal% carbohydrate) (#D20072301, Research Diets, Inc., New Brunswick, NJ, USA). Approximately half the animals on RD and MFD (Supplementary Figure S1) were aerosol infected with Mycobacterium tuberculosis HN878 strain using a full body Inhalation Exposure System (Glas-Col) in the ABSL-3 facility at CDI RAF at 8 weeks of age as previously described [29]. Briefly, *Mtb* aerosols were generated by a Glas-Col Inhalation Exposure System (Glas-Col) with a 5 mL bacterial suspension of about 3 × 10^6^ bacilli/mL in PBS containing 0.04% Tween 80, and the mice were exposed to the aerosol for 30 min, which results in seeding of approximately 100 colony forming units (CFU) per lung. Age- and sex-matched uninfected mice fed on either RD or MFD served as respective controls. All animals had ad libitum access to water and the respective rodent research diets (#D12450J or #D20072301). Both uninfected and *Mtb*-infected mice on RD were further randomized into two subgroups at 10 weeks of age and one subgroup was subjected to fat ablation (FAB+) by administering B/B Homodimerizer (#635058, Takara Bio, San Jose, CA, USA) intraperitoneally (ip) at a dose of 0.5 μg/g of body weight once daily for a total of 10 doses (Supplementary Figure S1). Mice were euthanized at 30 days post-infection (12 weeks of age) and samples including lungs, visceral adipose tissue, and spleen were harvested along with terminal blood collection. An equal number of uninfected mice per group per sex were included in the study design for a baseline comparison. A flowchart illustrating the experimental design is presented in Supplementary Figure S1. 

### 4.3. Mtb Colony Formation Assay

Portions of the freshly harvested left lung were homogenized in PBST using gentleMACS Octo Dissociator with M tubes (MiltenyiBiotec, Auburn, CA, USA). According to Ayyappan et al. 2018, homogenates were serially diluted and plated onto Difco Middlebrook 7H10 agar (BD Diagnostics, Franklin Lakes, NJ, USA) plates and incubated in a 37 °C incubator for 3 weeks to determine the number of bacterial CFU [45].

### 4.4. Histological Analyses

Freshly harvested lung lobes per mice were fixed with 10% neutral-buffered formalin for a minimum of 48 h and then embedded in paraffin wax and sectioned for histological analyses. Hematoxylin and eosin (H&E) staining was performed on sections of both uninfected and *Mtb*-infected lungs and the images were captured as previously described [29,60]. We used a small section of one lobe of the lung per mouse for histology. Five images (40× magnification) per section of each infected lung section were graded blindly by four investigators and the average scores were plotted as a dot plot. For each sample, the histological evidence of pulmonary pathology was classified in terms of the presence of infiltrated immune cells, loss of lipid droplets, and foamy macrophages and was graded on a 6-point scale ranging from 0 to 5 [9,10]. Point 0 indicates no abnormality, while points 1–5 represent increments of 20% each. Auramine-rhodamine (AR) staining of *Mtb*-infected lung sections was performed to detect and quantify the presence of *Mtb* burden, and the images were captured using fluorescence microscopy [10,29]. Immunohistochemistry (IHC) was performed on the formalin-fixed lung sections using rabbit polyclonal CD4 antibody (#NBP1-19371, Novus Biologicals, Centennial, CO, USA), rabbit polyclonal CD8 antibody (#NBP3-14871, Novus Biologicals, Centennial, CO, USA) and mouse monoclonal F4/80 antibody (#SC-377009, Santa Cruz Biotechnology, Dallas, TX, USA) with a dilution of 1:250, followed by a biotinylated secondary antibody using the VECTASTAIN Elite ABC-HRP kit (#PK-6101, Vector Laboratories, Newark, CA, USA). The antibody-stained sections were then washed and incubated with peroxidase substrate and counterstained with hematoxylin. Four to six images per section were captured and analyzed as previously described [29].

### 4.5. Immunoblot Analysis

Protein lysates from lungs were prepared by homogenizing the tissue using a handheld homogenizer after the addition of cell lysis buffer (#9803, Cell Signaling Technology, Danvers, MA, USA) containing Pierce protease inhibitor cocktail (#A32963, ThermoFisher Scientific, Waltham, MA USA). Uninfected and *Mtb*-infected samples were processed separately in BSL-2 and BSL-3 facilities, respectively. The homogenates were then incubated on ice for 10 min before clarification by centrifugation for 15 min at 14,000 rpm in a cold microfuge [29,60,61]. The supernatant was recovered and filtered through 0.22 µm spin filter tubes (only for infected samples), and the protein concentration was quantified using the Pierce BCA protein assay kit (#23225, ThermoFisher Scientific, Waltham, MA USA). We loaded 30 µg total protein from each sample and resolved it on SDS-PAGE, and then transferred the proteins onto a nitrocellulose membrane for immunoblot analysis. Primary antibodies against phospho-Perilipin 1 (ser 522) (#4856, Vala Sciences, San Diego, CA, USA), Cytochrome c (#4280, Cell Signaling Technology, Danvers, MA, USA), SOD1 (#4266, Cell Signaling Technology, Danvers, MA, USA), IFNγ (#MM701, Invitrogen, Waltham, MA, USA), IL-6 (#66146-1-Ig, Proteintech, Rosemont, IL, USA), IL-10 (#60269-1-Ig, Proteintech, Rosemont, IL, USA) and CHOP (#2895, Cell Signaling Technology, Danvers, MA, USA) were used to detect the expression of target proteins. Horseradish peroxidase (HRP)-conjugated anti-mouse immunoglobulin (#7076, Cell Signaling Technology, Danvers, MA, USA) and HRP-conjugated anti-rabbit immunoglobulin (#7074, Cell Signaling Technology, Danvers, MA, USA) were used as appropriate secondary antibodies to detect the chemiluminescent signal using Invitrogen iBright Imaging Systems. Guanosine nucleotide dissociation inhibitor (GDI) (#71-0300, Invitrogen, Waltham, MA, USA) was used as a loading control to normalize protein levels.

### 4.6. Tissue Dissociation and Cell Separation

Single-cell suspensions were made by dissociating freshly harvested *Mtb*-infected lung tissue using a lung dissociation kit (#130-095-927, MiltenyiBiotec, Auburn, CA, USA) in combination with the gentleMACS Octo Dissociator with heaters along with C tubes (MiltenyiBiotec) following the manufacturer’s instructions. All the infected tissue samples were processed in the ABSL-3 facility at CDI RAF. The single cell suspensions were then subjected to RBC lysis followed by MACS magnetic microbead-based cell isolation to separate and enrich CD4 (#130-116-475, MiltenyiBiotec, Auburn, CA, USA), CD8 (#130-116-478, MiltenyiBiotec, Auburn, CA, USA) and F4/80 (#130-110-443, MiltenyiBiotec, Auburn, CA, USA) populations using the MultiMACS Cell24 Separator Plus system according to the manufacturer’s instructions. Similarly, distinct CD4 and CD8 cell populations were isolated from uninfected spleen tissue using MACS microbeads in combination with the MultiMACS Cell24 Separator.

### 4.7. RNA Isolation and Quantitative PCR

Total host RNA from *Mtb*-infected lung tissue, *Mtb*-infected lung-derived CD8 cells and uninfected spleen-derived CD8 cells were isolated using a combination of TRIzol reagent (Invitrogen, Waltham, MA, USA) and the RNeasy Mini kit (Qiagen, Germantown, MD, USA). Isolated RNA samples were subjected to on column DNase I treatment to eliminate genomic DNA contamination. Purified RNA yield was assessed by a NanoDrop Microvolume Spectrophotometer (Thermo Fisher Scientific) and stored at −80 °C. Then 0.5-1 µg total RNA was reverse transcribed using the SuperScript VILO cDNA Synthesis Kit (#11754250, Invitrogen, Waltham, MA, USA) according to the manufacturer’s instructions. The cDNA samples were diluted at 1:5 and quantitative real-time PCR (qRT-PCR) experiments were performed using RT2 SYBR Green ROX Mastermix (#330523, Qiagen, Germantown, MD, USA) following the manufacturer’s instructions. The following primers were used for the target gene amplification: *M. tuberculosis SigA*, *FadA*, *Echs1*, *BfrA*, *Esat6*, *Cfp10*, *16s* and host *Tnfa*, *Ifng*, *Nadha*, *Sdhc*, *Cytob*, *Cox1a*, *18s*, *Hprt* (Supplementary Table S1). All assays were performed on an Applied Biosystems QuantStudio 3 Real-time PCR System according to the following cycling conditions: 10 min at 95 °C (1 cycle, HotStart DNA Taq Polymerase activation), followed by 15 s at 95 °C and 1 min at 60 °C (40 cycles, PCR amplification and data collection). Dissociation (melting) curve analysis was added to the run set up by enabling the default melting curve program to verify PCR specificity. Data analysis was performed normalized to the expression of 16 s (*Mtb* genes) and 18 s/HPRT (host genes) using the 2^−ΔΔCT^ method and the mRNA expression (fold change) levels were plotted as bar graphs. For each sample, both the housekeeping and target genes were amplified in triplicate [9].

### 4.8. Flow Cytometry Analysis

Single cell suspensions were prepared from freshly harvested *Mtb*-infected lung tissues and RBC lysed as described earlier. For immunophenotyping analysis of infiltrated immune cell populations, the dissociated lung cells from *Mtb*-infected animals (n = 3–4/sex/group) were stained for cell surface antigens with a fluorochrome-conjugated antibody cocktail comprising CD45-BV605, CD11b-APCCy7, F4/80-FITC, CD3-PE, NK1.1-PerCP-Cy5.5, CD4-BV421 and CD8a-PE-Cy7 followed by cell fixation using the BD Cytofix/Cytoperm kit (#554714, BD Biosciences, Franklin Lakes, NJ, USA) [62]. For analysis of cytokine production in T-lymphocytes, the cells were pooled in each group and treated with a cell stimulation cocktail (#00-4970-93, Invitogen, Waltham, MA, USA) in conjunction with BD GolgiStop (#554724, BD Biosciences, Franklin Lakes, NJ, USA) and BD GolgiPlug (#555029, BD Biosciences, Franklin Lakes, NJ, USA) protein transport inhibitors and incubated for 5 h at 37 °C. The stimulated cells were surface stained with a fluorochrome-conjugated antibody cocktail comprising TCRβ-BV711, CD4-BV421 and CD8a-APC-Cy7 followed by cell fixation and permeabilization. The surface-stained T-lymphocytes were divided into 2 fractions for intracellular cytokine staining, one fraction stained for IFNγ-PerCP-Cy5.5, TNFα-APC, IL-2-PE and the other stained for FOXP3-PerCP-Cy5.5, IL-4-APC, IL-17A-PE. Stained samples were analyzed on a BD LSRFortessa Cell Analyzer equipped with 4 lasers and 18 detectors. Acquisition and analyses were performed using BD FACSDiva and FlowJo software version 10.9 (Becton Dickinson, Franklin Lakes, NJ, USA).

### 4.9. Analysis of Circulatory Cytokines

Sandwich ELISA was performed to determine the levels of TNFα and IFNγ in blood plasma collected from uninfected and *Mtb*-infected male and female mice used in this study [9,29]. Samples were analyzed using mouse ELISA kits for TNFα (# BMS607-3, Invitrogen, Waltham, MA, USA) and IFNγ (# KMC4021, Invitrogen, Waltham, MA, USA) according to the manufacturer’s instructions. Quantitative analyses were performed on a Spark multimode microplate reader (TECAN).

### 4.10. Statistical Analysis

All statistical analyses were performed using GraphPad Prism version 10 (GraphPad Software, Boston, MA, USA). Data are presented as mean ± SEM. Comparisons between groups were analyzed using either Student’s *t*-test or two-way ANOVA, with sex, infection, fat-ablation, or fat gain as appropriate variables, as mentioned in the respective figure legends. *p*-values ≤ 0.05 were considered statistically significant between indicated groups.

## 5. Conclusions

In summary, our study highlights the sex-specific differences in the regulation of lipid metabolism, the distribution and activation of CD8^+^ T-cells, and mitochondrial functions in response to varying body fat levels during acute *Mtb* infection. Our comprehensive data also reveal notable variations in cytokine levels and proinflammatory signaling at systemic, localized, and immune cellular levels between males and females during *Mtb* infection.

## Figures and Tables

**Table 1:** Flow cytometry analysis of intracellular cytokine expression in infiltrated T-cells from *Mtb*-infected murine lung under PMA and ionomycin stimulation (using pooled samples).

		Male						Female					
		IFNγ	TNFα	IL-2	FOXP3	IL-4	IL-17A	IFNγ	TNFα	IL-2	FOXP3	IL-4	IL-17A
TCRβ^+^ CD4^+^	RD	4.43	68.4	3.14	1.05	0.77	1.13	14.2	46.8	2.67	0.62	0.23	2.45
	FAB+ RD	25.4	53.8	2.25	3.9	2.86	4.34	7.6	33.9	3.53	2.09	1.72	2.14
	MFD	13.5	43.1	1.31	1.5	0.6	1.63	15.8	53.8	2.83	1.08	0.81	3.58
TCRβ^+^ CD8^+^	RD	18.4	59.5	4.7	0.52	0.43	1	13.8	47.2	4.96	0.71	0.54	1.77
	FAB+ RD	15.1	52	2.9	0.62	0.15	1.08	8.31	34.2	3.31	0.99	1.34	1.91
	MFD	10	45.6	2.86	1.07	0.15	0.69	8.02	38.6	3.13	2.29	0.53	1.94

RD, regular diet; FAB+, fat ablated; MFD, medium-fat diet.

## Data Availability

Data are contained within the article or Supplementary Materials.

## Supplementary Materials

The following supporting information can be downloaded at: https://www.mdpi.com/article/10.3390/ijms25136823/s1.
