# Supplementary material for: The Influence of Body Fat Dynamics on Pulmonary Immune Responses in Murine Tuberculosis: Unraveling Sex-Specific Insights
Source: Int J Mol Sci. 2024 Jun 21;25(13):6823. doi: 10.3390/ijms25136823 (PMC11241512; doi:10.3390/ijms25136823)
Supplement: Supplementary file 1 [file ijms-25-06823-s001.zip › ijms-2979478-supplementary.pdf]

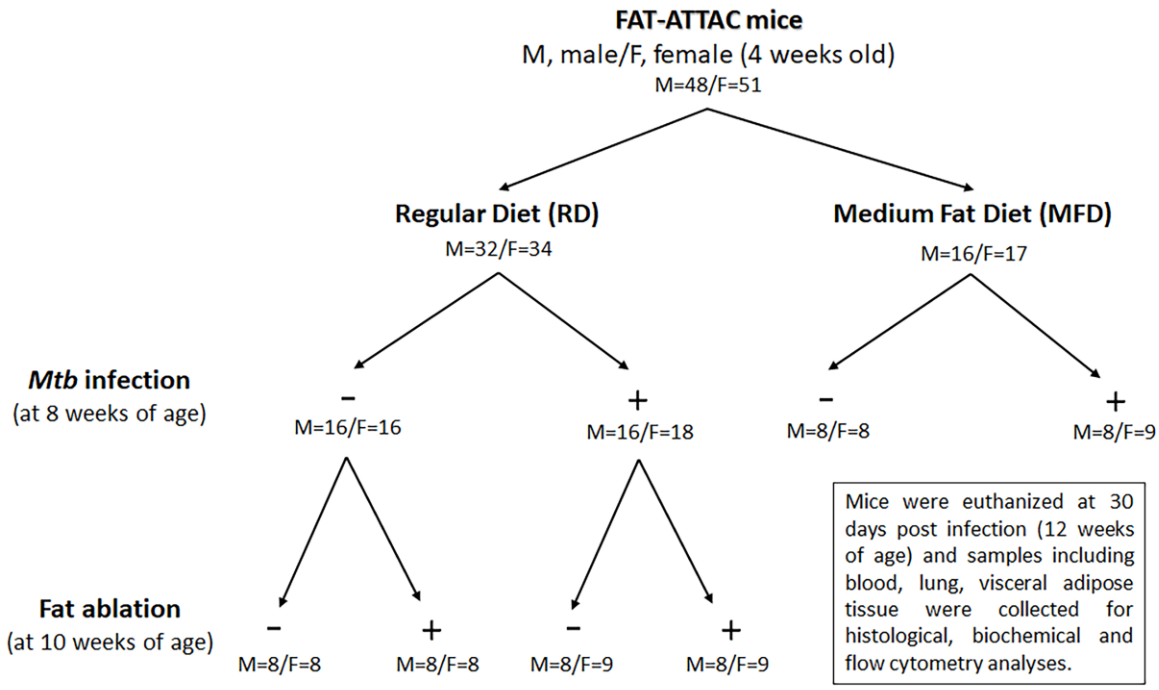

Figure S1. Flow chart illustrating the experimental design.

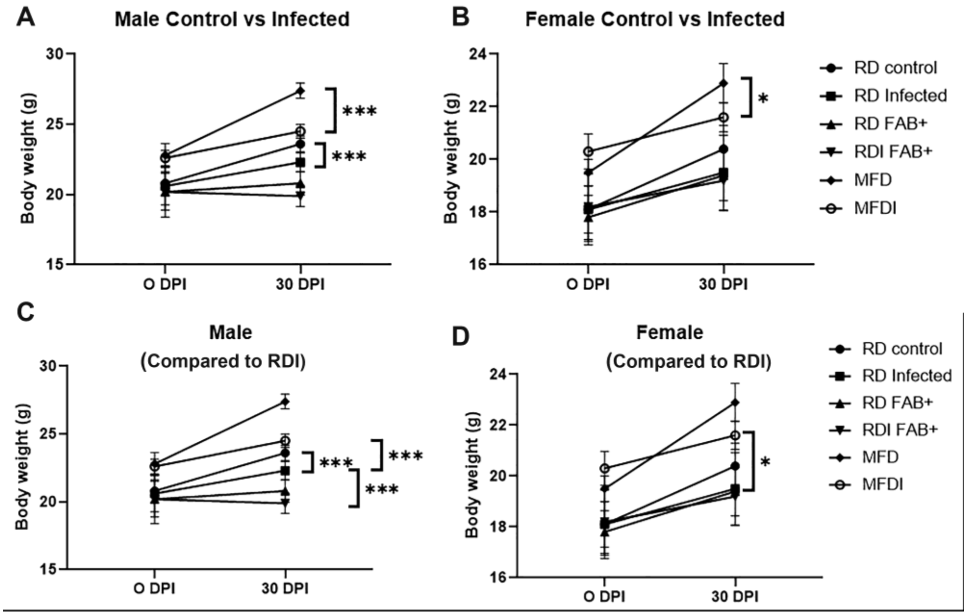

Figure S2. Body weight measurements reveal distinct alterations in body weights between male and female mice infected with *Mtb*, attributed to both fat loss and fat gain. (A-B) Line graphs depict variations in body weights from 0 to 30 DPI (Days Post-Infection) in male (A) and female (B) mice, contrasting with their respective counterparts: diet-fed or fat-ablated uninfected mice. (C-D) Additional line graphs illustrate the disparity in body weight changes among different infected groups, subjected to treatments for fat loss or fat gain. These findings are juxtaposed with infected male mice fed a regular diet (C) and infected female mice fed a regular diet (D). The error bars represent standard deviation of the mean. * $p < 0.05$, ** $p < 0.01$, and *** $p < 0.001$ between indicated groups.

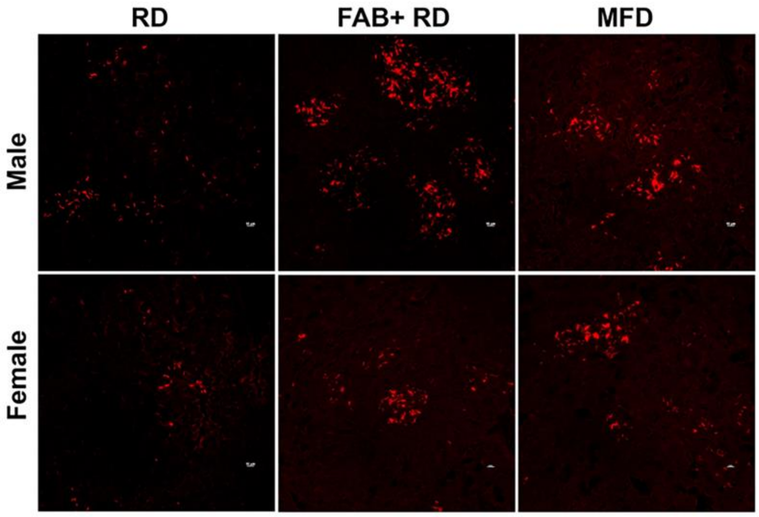

Figure S3. Auramine–Rhodamine staining of the lung sections showing the presence of *Mtb* (fluorescent red rods) in HN878 infected FAT-ATTAC mice.

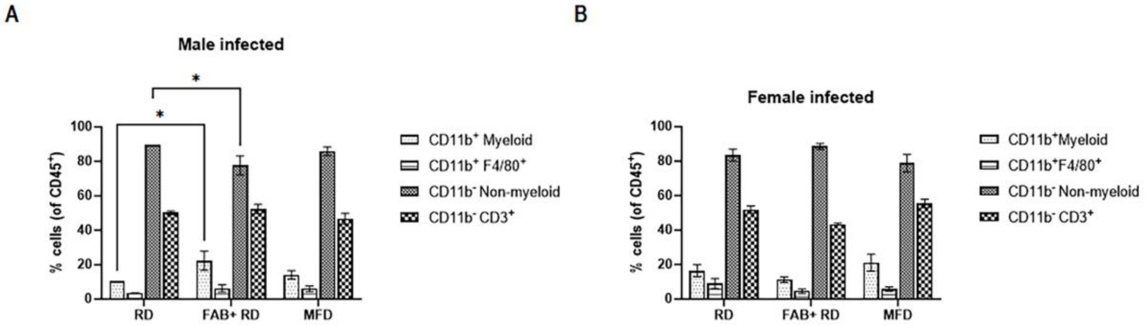

Figure S4. Flow cytometry analysis of *Mtb*-infected lungs showing infiltrated immune cell population in male and female mice. SuppleBar graph showing the percentage of $CD11b^+$ myeloid, $CD11b^+F4/80^+$ (double positive), $CD11b^-$ non-myeloid and $CD11b^-CD3^+$ cells from parent $CD45^+$ population among different groups in infected males (A) and females (B). The error bars represent standard error of the mean. * $p < 0.05$ between indicated groups.

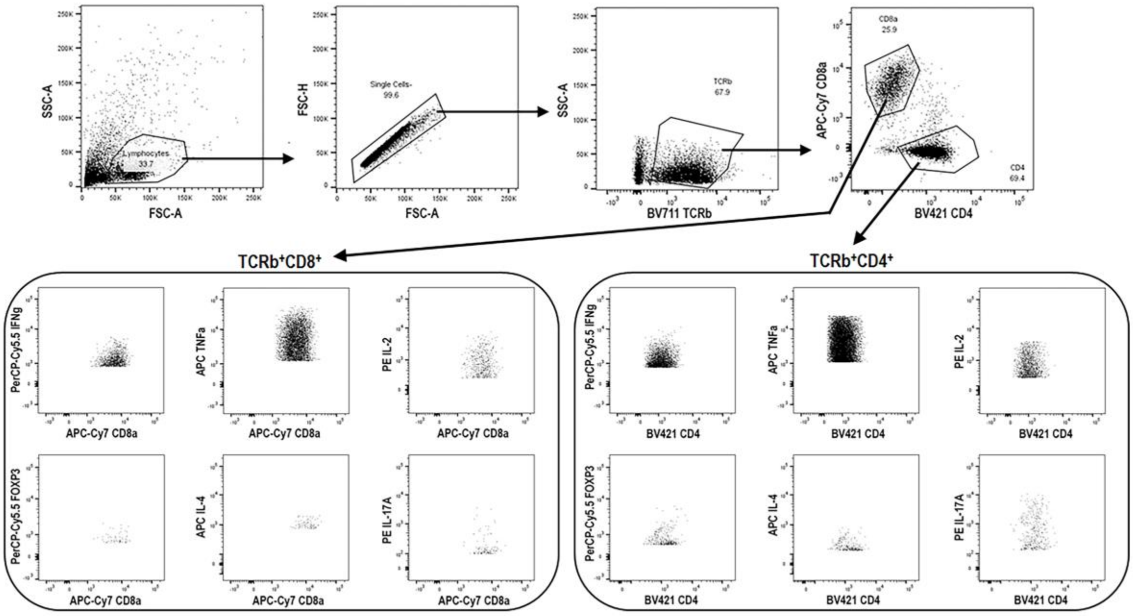

Figure S5. Gating strategy for flow cytometry analysis of cytokine production in T-lymphocytes in *Mtb*-infected murine lungs.

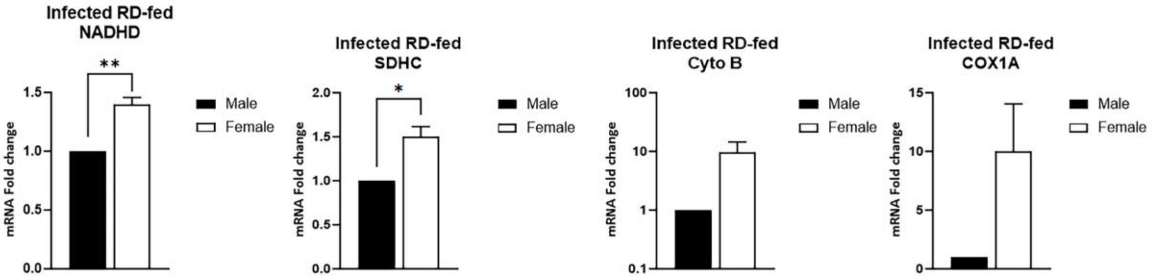

Figure S6. Quantitative PCR analysis of mitochondrial genes in naïve CD8+ T-cells between infected RD-fed male and female mice. The error bars represent standard error of the mean. * $p < 0.05$, and ** $p < 0.01$ between male and female; Table S1: Quantitative PCR primer sequences for *Mtb* genes and host genes analyzed in this study.
